# COVID-19 Infection Among Healthcare Workers: Serological Findings Supporting Routine Testing

**DOI:** 10.3389/fmed.2020.00471

**Published:** 2020-08-21

**Authors:** Ariel D. Stock, Edward R. Bader, Phillip Cezayirli, Julio Inocencio, Samantha A. Chalmers, Reza Yassari, Vijay Yanamadala, Emad Eskandar

**Affiliations:** ^1^Montefiore Medical Center Department of Neurological Surgery, Bronx, NY, United States; ^2^Albert Einstein College of Medicine Department of Neuroscience, Bronx, NY, United States; ^3^Albert Einstein College of Medicine Department of Microbiology and Immunology, Bronx, NY, United States

**Keywords:** COVID-19, SARS-CoV-2, healthcare worker, asymptomatic infection, coronavirus, asymptomatic infection carriers

## Abstract

A growing body of evidence demonstrates that asymptomatic and pre-symptomatic transmission of SARS-CoV-2 is a major contributor to the COVID-19 pandemic. Frontline healthcare workers in COVID-19 hotspots have faced numerous challenges, including shortages of personal protective equipment (PPE) and difficulties acquiring clinical testing. The magnitude of the exposure of healthcare workers and the potential for asymptomatic transmission makes it critical to understand the incidence of infection in this population. To determine the prevalence of asymptomatic SARS-CoV-2 infection amongst healthcare workers, we studied frontline staff working in the Montefiore Health System in New York City. All participants were asymptomatic at the time of testing and were tested by RT-qPCR and for anti-SARS-CoV-2 antibodies. The medical, occupational, and COVID-19 exposure histories of participants were recorded via questionnaires. Of the 98 asymptomatic healthcare workers tested, 19 (19.4%) tested positive by RT-qPCR and/or ELISA. Within this group, four (4.1%) were RT-qPCR positive, and four (4.1%) were PCR and IgG positive. Notably, an additional 11 (11.2%) individuals were IgG positive without a positive PCR. Two PCR positive individuals subsequently developed COVID-19 symptoms, while all others remained asymptomatic at 2-week follow-up. These results indicate that there is considerable asymptomatic infection with SARS-CoV-2 within the healthcare workforce, despite current mitigation policies. Furthermore, presuming that asymptomatic staff are not carrying SARS-CoV-2 is inconsistent with our results, and this could result in amplified transmission within healthcare settings. Consequently, aggressive testing regiments, such as testing frontline healthcare workers on a regular, multi-modal basis, may be required to prevent further spread within the workforce and to patients.

## Introduction

Throughout the progression of the COVID-19 pandemic, healthcare workers (HCWs) have experienced high levels of exposure to SARS-CoV-2, with the risk of infection rising with each time point of exposure (1, 2). HCWs are at greatest risk of SARS-CoV-2 infection, representing a large percentage of new infections. This, in part, has related to challenges in acquiring adequate personal protective equipment (3), resulting in a great deal of anxiety and distress amongst providers due to concern for self-infection with COVID-19 and family exposure (4). An important element in the discussion of community and healthcare-worker infection relates to asymptomatic and pre-symptomatic transmission of COVID-19, which may occur in up to 30% of individuals (5–7). New York City has been more severely affected then most (8), with widespread community infection, including a significant high-acuity disease burden (9). Attempts to address this concern are currently in their infancy, with widespread rollout of PCR-based and serological assessment in their early phases (10). Results of a recent New York State pilot study, which randomly tested 15,000 residents for serological evidence of SARS-CoV-2 exposure, found over 10% seroconversion statewide, with nearly 25% seropositivity in New York City (11). Importantly, we do not yet know the significance of seropositivity against SARS-CoV-2, particularly since most serological studies have been done in patients with a history of severe disease, and relative titers in asymptomatic carriers may not indicate immunity from transmission or infection (12).

Given the elevated risk of COVID-19 infection among HCWs and the consequent distress and concern from potential asymptomatic infection and transmission, we endeavored to address the rate of asymptomatic or possibly resolved infection among HCWs. We proceeded to test a cohort of clinicians at our institution for COVID-19 infection, including those working in COVID-19 intensive care units, specialty service physicians, and ambulatory staff. We evaluated both current infection via RT-qPCR sampling for SARS-CoV-2 and serology for the presence of anti-SARS-CoV-2-IgG antibodies. Beyond assessing the rate of active and resolving infections within our clinicians, internal testing would allow us to help prevent further spread of COVID-19 by serving as a screening tool, keeping any infected, asymptomatic HCWs quarantined pending disease presentation. Finally, the ability to reassure our HCWs that they are not infected and identify HCWs who may have silently recovered from COVID-19 is important for attenuating worker anxiety.

## Methods

### Study Design and Oversight

This cross-sectional study was approved by the Albert Einstein College of Medicine Institutional Review Board, with all subjects providing written informed consent. The goal of this study was to identify both asymptomatic HCW carriers of the SARS-CoV-2 virus, as well as those that may be immune to the virus, as denoted by serum IgG anti-SARS-CoV-2-nucleocapsid (IgG-anti-n) antibodies. These results would then assist in determining the safe deployment of staff within the hospital system to meet the demands of the COVID-19 healthcare crisis as well as provide an assessment of the rate of clinician infection in a COVID-19 hotspot.

### Study Participants/Demographics

Adult clinicians working within the Montefiore Health System, Bronx, New York City, active during the COVID-19 pandemic, were recruited to participate in the study (testing conducted between 04/04–20/2020). Three positive control samples (initially testing positive by RT-qPCR between 3/23–4/5/2020 and at least 2 weeks prior to serum sampling for serology) were included as well. The goal was to sample clinicians with varying degrees of hospital exposure to COVID-19 patients who were not exhibiting typical symptoms of COVID-19 (including fever, cough, and shortness of breath) at the time of participation. Exclusion criteria included an age over 65, as the risk of infection during testing outweighed the benefits, and individuals with any signs or symptoms typical of COVID-19. Each participant completed a survey pertaining to the current COVID-19 pandemic, exposure, workplace histories, recent history of symptoms attributable to COVID-19 infection, and medical history (used to calculate the Charlson Comorbidity Index score). Statistical relationships between groups were calculated using the Pearson's Chi-squared test and Fisher's Exact test for categorical variables, the Mann-Whitney-U test for continuous variables, and the Kruskal Wallis H test for comparison of IgG titers.

### SARS-CoV-2 RT-qPCR Testing

Participants underwent both nasopharyngeal and oropharyngeal swabbing concurrently, and samples were pooled. We collected swabs directly into the RNA-lysis buffer, and we then isolated RNA using a Zymo Research RNA MicroKit (Irvine, CA) according to manufacturer's recommendations. Each RNA sample was evaluated by spectrophotometry and then analyzed by RT-qPCR according to CDC-recommended protocols (13) for SARS-CoV-2 testing with slight modification, utilizing primers to the *nucleocapsid* gene (N1 and N2) and *RnaseP* (RP) as a control (IDTDNA, Coralville, IA). Commercially available plasmid controls were utilized for all primer sequences (IDTDNA). After validating accuracy on several positive controls and redundantly running the reaction on multiple samples, the reaction volume was scaled down from a 96-well-plate format to a 384-well-plate format, with samples run on the Applied Biosystems Via7 system and analyzed using the QuantStudio software package (Thermo Scientific, Waltham, MA).

### ELISA for Anti-SARS-CoV-2-Nucleocapsid IgG

Blood was collected from each participant into serum separator tubes (BD, Franklin Lakes, NJ), allowed to coagulate at room temperature for 60 min, and then stored at 4°C until centrifugation. Serum was analyzed in duplicate using an anti-n IgG ELISA (Epitope Diagnostics Inc., San Diego, CA), according to manufacturer's recommendations with slight modification. Assay cut-off values per the protocol were determined as follows: the optical densities of the negative control samples (all of which between 0.19 and 0.22) were averaged and adjusted by addition of a constant (0.18). This resultant reference value was then multiplied by a correction factor of 1.1 (which represents the cutoff value); anything above this being positive and anything below being negative. In addition to the internal controls provided with the kit, we included three participants with a history of RT-qPCR-positive SARS-CoV-2 infection as positive controls.

### Performance of Clinically Administered SARS-CoV-2 Testing

To assess the performance of clinically administered testing, biostatistics were calculated by comparing hospital-administered RT-qPCR testing with the anti-n IgG ELISA testing we employed, using anti-n IgG ELISA as the reference standard for historical infection in this case. Only individuals whose clinically administered RT-qPCR test occurred ≥14 days before anti-n IgG ELISA testing were included to allow time for a detectable IgG antibody response to develop. Sensitivity, specificity, positive predictive value, negative predictive value, and accuracy were calculated alongside 95% confidence intervals.

## Results

### Subject Characteristics

We evaluated 98 clinicians working in the Montefiore Health System who have been clinically active since the early part of the COVID-19 pandemic within New York City. Several work environments were represented, including COVID-19 medicine units, COVID-19 ICUs, the ED, specialty consultants, and those working in a purely ambulatory setting. These individuals had varying degrees of workplace exposure to COVID-19 patients, including invasive bedside procedures with COVID-19-positive patients, intraoperative exposure, as well as in routine care. Interestingly, overall exposure histories were not correlated with testing results (*p* = 0.292, Table 1). Additionally, a history of COVID-19-like illness was not correlated with optical densities on ELISA (*p* = 0.112, Table 2). Importantly, none of the subjects were symptomatic at the time of testing, though some were previously tested due to exposure and/or typical COVID-19 symptoms.

**Table 1:** Demographics and clinical characteristics of frontline healthcare providers tested for SARS-CoV-2.

**Demographics and clinical variables**	**Total (*n* = 98)**	**Positive^a^ (*n* = 19)**	**Negative (*n* = 79)**	***P*-value^b^**
Mean age (+/– SD)- yr.	37.6 (10.6)	38.8 (13.7)	37.4 (9.8)	0.815^1^
Sex—no. (%)				1.000^2^
Male	49 (50.0)	10 (52.6)	39 (49.4)	
Female	49 (50.0)	9 (47.4)	40 (50.6)	
Job Type—no. (%)				0.240^3^
Both inpatient and outpatient	86 (87.8)	15 (79.0)	71 (89.9)	
Exclusively outpatient	12 (12.3)	4 (21.5)	8 (10.1)	
Job Title—no. (%)				0.415^3^
Physician	62 (63.3)	12 (63.2)	50 (63.3)	
Physician's Assistant	15 (15.3)	4 (21.5)	11 (13.9)	
Nurse Practitioner	9 (9.2)	3 (15.8)	6 (7.6)	
Nurse	6 (6.1)	0	6 (7.6)	
Perfusionist	6 (6.1)	0	6 (7.6)	
SARS-CoV-2 Exposure Risk Index- no. (%)				0.292^3^
No known exposure	7 (7.1)	1 (5.3)	6 (7.6)	
Wearing full PPE	57 (58.2)	8 (42.1)	49 (62.0)	
Conventional droplet precautions	16 (16.3)	5 (26.3)	11 (13.9)	
No PPE	18 (18.4)	5 (26.3)	13 (16.5)	
Typical COVID-19 symptoms- no. (%)				0.052^2^
Absent	67 (68.4)	9 (47.3)	58 (73.4)	
Present	31 (31.6)	10 (52.6)	21 (26.6)	
Comorbidities—no. (%)				0.785^2^
None	68 (69.4)	14 (73.7)	54 (68.4)	
Asthma	10 (10.2)	2 (10.5)	8 (10.1)	
Hypertension	5 (5.1)	2 (10.5)	3 (3.8)	
Hyperlipidemia	5 (5.1)	0	5 (6.3)	
Malignancy	4 (4.1)	1 (5.3)	3 (3.8)	
Autoimmune disease	3 (3.1)	0	3 (3.8)	
Diabetes mellitus	2 (2.0)	1 (5.3)	1 (1.3)	
Inflammatory bowel disease	2 (2.0)	0	2 (2.5)	
Endocrine disorder	2 (2.0)	0	2 (2.5)	
Hematological disorder	2 (2.0)	0	2 (2.5)	
Charlson Comorbidity Index – no. (%)				0.205^3^
0	80 (81.6)	13 (68.4)	67 (84.0)	
1	10 (10.2)	4 (21.1)	6 (7.6)	
2	4 (4.1)	1 (5.3)	3 (3.8)	
3	1 (1.0)	0	1 (1.3)	
4	3 (3.1)	1 (5.3)	2 (2.5)	

a *Defined as a positive swab PCR and/or positive serum IgG ELISA*.

b P-values calculated using

1 Mann-Whitney U test, Pearson's

2 Chi-squared test, or

3 *Fisher's exact test*.

**Table 2:** IgG ELISA optical densities of frontline healthcare workers tested for SARS-CoV-2.

**SARS-CoV-2 test results**	**Total (*N* = 98)**	**Mean IgG ELISA OD (+/– SEM)**	***P*-value^a^**
Result profile groups			<0.001^1^
PCR Positive—no. (%)	4 (4.1)	0.251 (0.032)	
PCR/IgG Positive—no. (%)	4 (4.1)	0.656 (0.055)	
IgG Positive—no. (%)	11 (11.2)	0.589 (0.081)	
PCR/IgG Negative—no. (%)	79 (80.6)	0.231 (0.008)	
IgG results by prior symptom profile			0.112^2^
No history of typical Covid symptoms—no. (%)	67 (68.4)	0.264 (0.016)	
Previous history of Covid symptoms—no. (%)	31 (31.6)	0.343 (0.043)	

a P-values calculated using

1 Kruskal-Wallis H test when comparing multiple groups, and

2 *Mann-Whitney U test*.

### RT-qPCR for Active Infection

**Figure 1:**
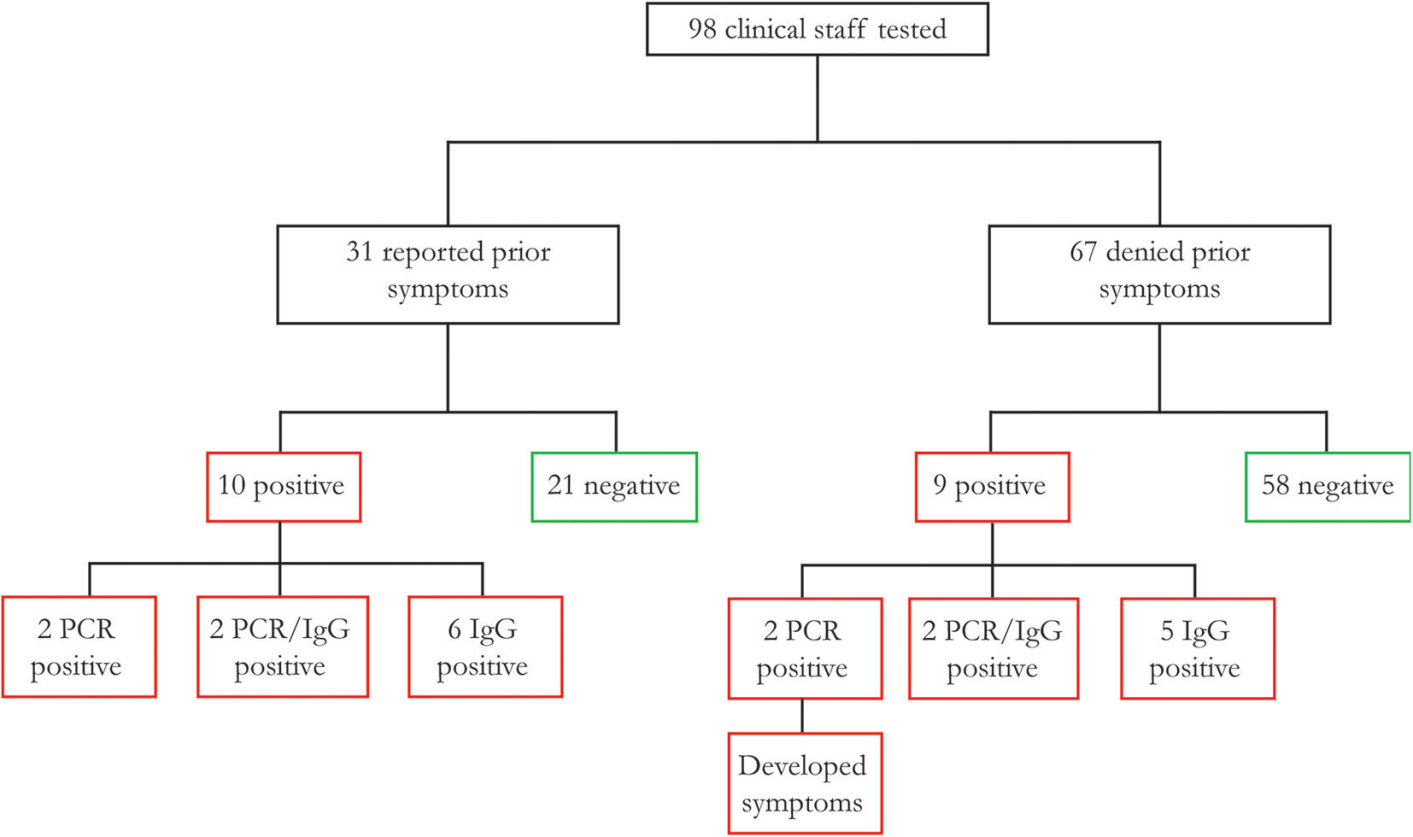
Association between prior complaint of COVID-19 symptoms and SARS-CoV-2 infection of 98 healthcare worker participants, 31 reported typical COVID-19 symptoms prior to study participation, of whom 32.3% were found to have a history of SARS-CoV-2 infection (as defined by RT-qPCR or serology). There were an additional nine participants with no symptoms prior to study participation, of whom two subsequently developed symptoms (PCR positive), and with others asymptomatic as of this publication.

Nasopharyngeal and oropharyngeal swabs underwent RNA purification, and spectrophotometry revealed excellent RNA yields and quality. RT-qPCR was run with 10 ng RNA per reaction in triplicates for each primer (N1, N2, and RP). All samples demonstrated RP expression, indicating adequate RNA isolation from respiratory epithelium. Through viral RNA amplification, we identified a total of eight individuals who were SARS-CoV-2 positive (PCR positive, 8% of tested clinical staff, Ct values are the average of triplicates for each positive sample, standard deviations of all triplicates <1, subjects 1–4 and 8–11, Figure 1, Table S1), including four with a history of resolved symptoms and two who subsequently developed moderate symptoms. Two other individuals noted vague upper respiratory symptoms in retrospect but were otherwise asymptomatic.

### Serum Anti-SARS-CoV-2-Nucleocapsid IgG

For serum evaluation, we focused on IgG for two reasons: it represents a more predictable and durable immune response than IgM and, once positive, should persist so for an extended period (14, 15). From a technical perspective, IgG is more readily and specifically assayable given its higher affinity for individual antigens (16). On ELISA, the positive control individuals, who had typical COVID-19 symptoms that resolved over 14 days prior to participation, had optical densities (ODs) above the positive threshold (0.560, 1.494, 1.166; PCR/IgG positive, subjects 5–7, Table S1), which is consistent with the literature on IgG responses during this pandemic (15). We further identified 15 individuals who met criteria for seropositivity (IgG positive, subjects 8–22, Table S1). Of the IgG positive group, four individuals were RT-qPCR positive (PCR/IgG positive, subjects 8–11) and 11 RT-qPCR negative (IgG positive, subjects 12–22). Interestingly, of the four PCR/IgG-positive individuals, two had a history of symptoms but were unable to secure testing due to limited availability. Among the 11 IgG-positive individuals, four had a history of symptoms with negative test results.

A total of 19 individuals had a history of clinically administered SARS-CoV-2 testing by RT-qPCR ≥14 days prior to participation in this study and anti-n IgG ELISA testing (Table S1). Of these 19, three tested positive on clinically administered testing; however, seven of these individuals tested positive by anti-n IgG ELISA in this study. When evaluating all those with a history of testing prior to this study, and assuming the validity of ELISA as the reference standard for history of infection (i.e., IgG positive = Infectious History), the sensitivity of clinically administered RT-qPCR based diagnostics for SARS-CoV-2 was only 42.86% (true positives/true positives + false negatives: 3/7 individuals), though importantly the specificity was 100% (true negatives/true negatives + false positives: 12/12 individuals) (Table 3). Subject #1 was characterized as a true negative, presumably having contracted SARS-CoV-2 in the interim.

**Table 3:** Sensitivity and specificity of clinically administered RT-qPCR testing prior to study participation (using anti-n IgG ELISA as the reference standard).

**Statistic**	**Value (*n*/*n* individuals)**	**95% CI**
Sensitivity	42.86% (3/7)	9.90–81.59%
Specificity	100% (12/12)	73.54–100.00%
Positive predictive value	100% (3/3)	
Negative predictive value	75.00% (12/16)	61.23–85.07%
Accuracy	78.95%	54.43–93.95%

*Sensitivity and specificity of clinically administered RT-qPCR testing for SARS-CoV-2 infection prior to participation in this study was calculated to include false negatives determined by current serology testing. Anti-n IgG ELISA was used as the reference standard. Subject #1 (Table S1) may represent a false negative or may have contracted the virus subsequent to clinical testing and was consequently characterized as a true negative*.

## Discussion

There has been extensive discussion among healthcare providers, researchers, and policy makers about the role that asymptomatic, undiagnosed infections play in the spread of SARS-CoV-2 (7). Additionally, HCWs have found themselves inadequately supplied with personal protective equipment while caring for COVID-19 patients (3). Finally, the types of social distancing, which, at the time of this writing, are having a successful impact on decreasing community SARS-CoV-2 spread, are not practically feasible within the healthcare work environment. Consequently, it is reasonable to expect an increased incidence of SARS-CoV-2 infection among healthcare workers. In this study, of the 98 asymptomatic healthcare workers tested, we identified 19 (19.4%) SARS-CoV-2-positive participants, as defined by PCR and/or serology.

Addressing the impact of SARS-CoV-2 among HCWs requires identifying those in the pre-symptomatic/asymptomatic phase as well as those who may have had the infection and may now be at an attenuated risk of infection or transmission. In a recent publication evaluating the SARS-CoV-2 transmission pattern in an early Washington State skilled nursing facility cluster, the authors highlighted the role that asymptomatic and/or pre-symptomatic transmission between residents certainly played in disease dissemination (7, 17). Furthermore, their findings support the inadequacy of relying on symptomatic presentation as the indicator for testing healthcare providers. The latter finding is consistent with our own in which more than 10% of asymptomatic HCWs presented with SARS-CoV-2 profiles consistent with either recent infection or seroconversion. Institutional and national testing limitations, meanwhile, represented a problem at the start of this pandemic, though routine screening of HCWs is recently available. Given the limited ability to test minimally symptomatic individuals during the early months of the pandemic, policymakers have largely suggested identifying those that have recovered, through serology, as an element of return to societal function in the future. The independent testing presented herein utilized both approaches, presenting important findings both regarding the infectious status of healthcare workers, as well as issues with PCR-based testing sensitivity, owing to fluctuating viral loads and sampling technique variability. Additionally, as the vast majority of testing has been validated in symptomatic individuals, the sensitivity of PCR in asymptomatic individuals, such as the ones studied here, remains uncertain.

We identified 19 of 98 clinicians (19.4%) that demonstrated either a new diagnosis (PCR positive) or a history of SARS-CoV-2 (IgG positive). These included four (4.1%) PCR-positive, four (4.1%) PCR/IgG-positive, and 11 (11.2%) PCR-negative/IgG-positive individuals. Of the 19 SARS-CoV-2 positive participants, 10 (10.2%) reported a prior history of COVID-19 symptoms, now presenting as two PCR-positive, two PCR/IgG-positive, and six IgG-positive individuals (Figure 1, Table 1). Of the nine participants without a prior history of COVID-19 symptoms, two were PCR-positive individuals that subsequently developed symptoms with an additional two PCR/IgG-positive and five IgG-positive participants that remained asymptomatic for 2 weeks after testing. Among all 16 (16.3%) participants with negative clinical testing for SARS-CoV-2 prior to study participation, four (4/16, 25%) were found to be IgG positive (suggesting prior false negative testing), and one (1/16, 6.3%) was PCR positive.

The IgG testing poses the primary limitation in this study or any of its kind at this time. While the assay has reportedly undergone validation with positive and negative controls, is accompanied by internal controls, and has demonstrated positivity with our own control participants, the novelty of available assays requires careful interpretation. For example, the recombinant nucleocapsid protein used in the chosen assays shares some degree of homology with other coronaviruses, including some, to which the studied population are routinely exposed (18, 19). Additionally, the cutoffs for positivity and negativity in the study, while reportedly validated by the supplier on positive control and pre-pandemic sera, leave an interval in between positive and negative results that are unclear (which we considered negative for the purposes of this study). Other assays, which use total viral lysate as a plated antigen, would pose similar challenges. Recent developments focused on a modified Spike protein, which appears to have improved *in-vitro* stability, as well as a more specific binding affinity for SARS-CoV-2, are underway as well (20). Most importantly, we have yet to determine whether seroconversion confers longstanding, seasonal, or limited immunity, making serology of limited, diagnostic utility at this time (12).

Despite inherent limitations in newly developed serological assays and their interpretation, RT-qPCR behaved as expected. A number of individuals were found to be persistently PCR positive, after an extended period of time from symptom onset, consistent with reports elsewhere (21–24). This feature of COVID-19 has the ancillary benefit of lending confidence to our IgG results, as there was concordance between testing results in nearly 40% of IgG positive individuals. The most significant of our findings, in line with the primary goal of this study, was *de novo* identification of eight asymptomatic individuals amongst clinicians that were PCR positive for SARS-CoV-2. This represents critical information in terms of staff management and deployment. Indeed, several participant HCWs, functioning in essential settings on the frontline of patient care, were pulled from active duty shortly prior to developing symptoms.

A better understanding of the dynamics of healthcare-worker infection with SARS-CoV-2 is essential in protecting this key element of the workforce as well as mitigating their role in nosocomial and community spread of COVID-19. While this study represents a limited snapshot, it identifies several important findings. Foremost, healthcare workers may be carrying and, therefore, spreading SARS-CoV-2, without any signs or symptoms of disease. Additionally, prior negative testing by PCR does not preclude infection, which can be identified serologically. We applaud government efforts to scale-up serological and PCR-based testing programs, but we caution that an individual timepoint may not provide adequate mitigation of SARS-CoV-2 transmission by and between healthcare workers. In future studies, it will be interesting to evaluate HCWs over time, to determine the rate of infection in a group with known regular exposure. Collectively, our findings suggest that it is appropriate to regularly test all healthcare workers in high disease burden areas for SARS-CoV-2 by both PCR and serological assays, irrespective of ostensible exposure or symptom history.

## Data Availability Statement

The raw data supporting the conclusions of this article will be made available by the authors, without undue reservation.

## Ethics Statement

The studies involving human participants were reviewed and approved by Albert Einstein College of Medicine IRB. The patients/participants provided their written informed consent to participate in this study.

## Author Contributions

AS conceived this study, executed most of the testing, and wrote much of this manuscript. EB performed data analysis, assisted with manuscript writing and data interpretation. PC assisted with data collection and analysis. JI assisted with sample collection and processing. SC assisted with study design and sample processing. RY assisted in data interpretation. VY helped design this study. EE was involved with the conception, design, and interpretation of this study. All authors contributed to the article and approved the submitted version.

## Conflict of Interest

The authors declare that the research was conducted in the absence of any commercial or financial relationships that could be construed as a potential conflict of interest.
