# Supplementary material for: COVID-19 Infection Among Healthcare Workers: Serological Findings Supporting Routine Testing
Source: Front Med (Lausanne). 2020 Aug 21;7:471. doi: 10.3389/fmed.2020.00471 (PMC7472984; doi:10.3389/fmed.2020.00471)
Supplement: Supplementary file 1 [file Table_1.docx]

Table S1. Individual results by participant.

| **ID** | **Covid-19 History Prior to Study  (Symptomatic, NEG/POS/NO PCR)** | **PCR  Status** | **Serology** | **Mean IgG  O.D.** | **Profile Groups** | **Ct N1** | **Ct N2** | **Ct RP** |
| --- | --- | --- | --- | --- | --- | --- | --- | --- |
| 1 | Sx/NEG PCR | + | - | 0.252 | PCR Positive | 33.05 | 32.72 | 23.02 |
| 2 | Sx/NO PCR | + | - | 0.340 | PCR Positive | 15.63 | 15.74 | 22.92 |
| 3 | - | + | - | 0.221 | PCR Positive | 34.14 | 35.60 | 23.49 |
| 4 | - | + | - | 0.189 | PCR Positive | 32.43 | 32.36 | 24.26 |
| 5 | Sx/POS PCR (Positive Control) | + | + | 0.560 | PCR/IgG Positive | 31.74 | 33.28 | 23.63 |
| 6 | Sx/POS PCR (Positive Control) | + | + | 1.494 | PCR/IgG Positive | 34.40 | 33.00 | 24.21 |
| 7 | Sx/POS PCR (Positive Control) | + | + | 1.166 | PCR/IgG Positive | 30.11 | 32.99 | 20.85 |
| 8 | Sx/NO PCR | + | + | 0.580 | PCR/IgG Positive | 25.47 | 25.86 | 24.30 |
| 9 | Sx/NO PCR | + | + | 0.579 | PCR/IgG Positive | 34.64 | 36.74 | 27.36 |
| 10 | - | + | + | 0.814 | PCR/IgG Positive | 27.78 | 30.66 | 20.93 |
| 11 | - | + | + | 0.652 | PCR/IgG Positive | 33.84 | 36.97 | 21.59 |
| 12 | Sx/NEG PCR | - | + | 1.229 | IgG Positive |  |  |  |
| 13 | Sx/NEG PCR | - | + | 0.995 | IgG Positive |  |  |  |
| 14 | Sx/NEG PCR | - | + | 0.507 | IgG Positive |  |  |  |
| 15 | Sx/NEG PCR | - | + | 0.486 | IgG Positive |  |  |  |
| 16 | Sx/NO PCR | - | + | 0.441 | IgG Positive |  |  |  |
| 17 | Sx/NO PCR | - | + | 0.397 | IgG Positive |  |  |  |
| 18 | - | - | + | 0.560 | IgG Positive |  |  |  |
| 19 | - | - | + | 0.508 | IgG Positive |  |  |  |
| 20 | - | - | + | 0.489 | IgG Positive |  |  |  |
| 21 | - | - | + | 0.453 | IgG Positive |  |  |  |
| 22 | - | - | + | 0.410 | IgG Positive |  |  |  |
| 23 | Sx/NEG PCR | - | - | 0.280 | PCR/IgG Negative |  |  |  |
| 24 | Sx/NEG PCR | - | - | 0.249 | PCR/IgG Negative |  |  |  |
| 25 | Sx/NEG PCR | - | - | 0.211 | PCR/IgG Negative |  |  |  |
| 26 | Sx/NEG PCR | - | - | 0.202 | PCR/IgG Negative |  |  |  |
| 27 | Sx/NEG PCR | - | - | 0.104 | PCR/IgG Negative |  |  |  |
| 28 | NEG PCR | - | - | 0.386 | PCR/IgG Negative |  |  |  |
| 29 | NEG PCR | - | - | 0.376 | PCR/IgG Negative |  |  |  |
| 30 | NEG PCR | - | - | 0.315 | PCR/IgG Negative |  |  |  |
| 31 | NEG PCR | - | - | 0.298 | PCR/IgG Negative |  |  |  |
| 32 | NEG PCR | - | - | 0.268 | PCR/IgG Negative |  |  |  |
| 33 | NEG PCR | - | - | 0.223 | PCR/IgG Negative |  |  |  |
| 34 | Sx/NO PCR | - | - | 0.328 | PCR/IgG Negative |  |  |  |
| 35 | Sx/NO PCR | - | - | 0.307 | PCR/IgG Negative |  |  |  |
| 36 | Sx/NO PCR | - | - | 0.297 | PCR/IgG Negative |  |  |  |
| 37 | Sx/NO PCR | - | - | 0.287 | PCR/IgG Negative |  |  |  |
| 38 | Sx/NO PCR | - | - | 0.281 | PCR/IgG Negative |  |  |  |
| 39 | Sx/NO PCR | - | - | 0.280 | PCR/IgG Negative |  |  |  |
| 40 | Sx/NO PCR | - | - | 0.256 | PCR/IgG Negative |  |  |  |
| 41 | Sx/NO PCR | - | - | 0.233 | PCR/IgG Negative |  |  |  |
| 42 | Sx/NO PCR | - | - | 0.231 | PCR/IgG Negative |  |  |  |
| 43 | Sx/NO PCR | - | - | 0.224 | PCR/IgG Negative |  |  |  |
| 44 | Sx/NO PCR | - | - | 0.218 | PCR/IgG Negative |  |  |  |
| 45 | Sx/NO PCR | - | - | 0.205 | PCR/IgG Negative |  |  |  |
| 46 | Sx/NO PCR | - | - | 0.198 | PCR/IgG Negative |  |  |  |
| 47 | Sx/NO PCR | - | - | 0.188 | PCR/IgG Negative |  |  |  |
| 48 | Sx/NO PCR | - | - | 0.131 | PCR/IgG Negative |  |  |  |
| 49 | Sx/NO PCR | - | - | 0.125 | PCR/IgG Negative |  |  |  |
| 50 | - | - | - | 0.324 | PCR/IgG Negative |  |  |  |
| 51 | - | - | - | 0.300 | PCR/IgG Negative |  |  |  |
| 52 | - | - | - | 0.294 | PCR/IgG Negative |  |  |  |
| 53 | - | - | - | 0.254 | PCR/IgG Negative |  |  |  |
| 54 | - | - | - | 0.198 | PCR/IgG Negative |  |  |  |
| 55 | - | - | - | 0.120 | PCR/IgG Negative |  |  |  |
| 56 | - | - | - | 0.379 | PCR/IgG Negative |  |  |  |
| 57 | - | - | - | 0.331 | PCR/IgG Negative |  |  |  |
| 58 | - | - | - | 0.325 | PCR/IgG Negative |  |  |  |
| 59 | - | - | - | 0.318 | PCR/IgG Negative |  |  |  |
| 60 | - | - | - | 0.313 | PCR/IgG Negative |  |  |  |
| 61 | - | - | - | 0.309 | PCR/IgG Negative |  |  |  |
| 62 | - | - | - | 0.293 | PCR/IgG Negative |  |  |  |
| 63 | - | - | - | 0.290 | PCR/IgG Negative |  |  |  |
| 64 | - | - | - | 0.289 | PCR/IgG Negative |  |  |  |
| 65 | - | - | - | 0.286 | PCR/IgG Negative |  |  |  |
| 66 | - | - | - | 0.279 | PCR/IgG Negative |  |  |  |
| 67 | - | - | - | 0.276 | PCR/IgG Negative |  |  |  |
| 68 | - | - | - | 0.272 | PCR/IgG Negative |  |  |  |
| 69 | - | - | - | 0.270 | PCR/IgG Negative |  |  |  |
| 70 | - | - | - | 0.268 | PCR/IgG Negative |  |  |  |
| 71 | - | - | - | 0.265 | PCR/IgG Negative |  |  |  |
| 72 | - | - | - | 0.254 | PCR/IgG Negative |  |  |  |
| 73 | - | - | - | 0.247 | PCR/IgG Negative |  |  |  |
| 74 | - | - | - | 0.236 | PCR/IgG Negative |  |  |  |
| 75 | - | - | - | 0.235 | PCR/IgG Negative |  |  |  |
| 76 | - | - | - | 0.231 | PCR/IgG Negative |  |  |  |
| 77 | - | - | - | 0.220 | PCR/IgG Negative |  |  |  |
| 78 | - | - | - | 0.213 | PCR/IgG Negative |  |  |  |
| 79 | - | - | - | 0.201 | PCR/IgG Negative |  |  |  |
| 80 | - | - | - | 0.197 | PCR/IgG Negative |  |  |  |
| 81 | - | - | - | 0.189 | PCR/IgG Negative |  |  |  |
| 82 | - | - | - | 0.188 | PCR/IgG Negative |  |  |  |
| 83 | - | - | - | 0.188 | PCR/IgG Negative |  |  |  |
| 84 | - | - | - | 0.185 | PCR/IgG Negative |  |  |  |
| 85 | - | - | - | 0.177 | PCR/IgG Negative |  |  |  |
| 86 | - | - | - | 0.173 | PCR/IgG Negative |  |  |  |
| 87 | - | - | - | 0.171 | PCR/IgG Negative |  |  |  |
| 88 | - | - | - | 0.170 | PCR/IgG Negative |  |  |  |
| 89 | - | - | - | 0.163 | PCR/IgG Negative |  |  |  |
| 90 | - | - | - | 0.161 | PCR/IgG Negative |  |  |  |
| 91 | - | - | - | 0.147 | PCR/IgG Negative |  |  |  |
| 92 | - | - | - | 0.146 | PCR/IgG Negative |  |  |  |
| 93 | - | - | - | 0.142 | PCR/IgG Negative |  |  |  |
| 94 | - | - | - | 0.140 | PCR/IgG Negative |  |  |  |
| 95 | - | - | - | 0.140 | PCR/IgG Negative |  |  |  |
| 96 | - | - | - | 0.137 | PCR/IgG Negative |  |  |  |
| 97 | - | - | - | 0.134 | PCR/IgG Negative |  |  |  |
| 98 | - | - | - | 0.133 | PCR/IgG Negative |  |  |  |
| 99 | - | - | - | 0.125 | PCR/IgG Negative |  |  |  |
| 100 | - | - | - | 0.120 | PCR/IgG Negative |  |  |  |
| 101 | - | - | - | 0.115 | PCR/IgG Negative |  |  |  |
